# Meanings and Experiences of Prisoners and Family Members Affected by the COVID-19 Pandemic in a Brazilian Prison Unit: A Grounded Theory Analysis

**DOI:** 10.3390/ijerph20156488

**Published:** 2023-08-01

**Authors:** Wanessa Cristina Baccon, Maria Aparecida Salci, Lígia Carreira, Adriana Martins Gallo, Francielle Renata Danielli Martins Marques, Marcelle Paiano, Vanessa Denardi Antoniassi Baldissera, Carlos Laranjeira

**Affiliations:** 1 Departamento de Pós-Graduação em Enfermagem, Universidade Estadual de Maringá, Avenida Colombo, 5790—Campus Universitário, Maringá 87020-900, PR, Brazil; wanessabaccon@hotmail.com (W.C.B.); masalci@uem.br (M.A.S.); ligiacarreira.uem@gmail.com (L.C.); adrianagallo.particular@gmail.com (A.M.G.); franrenata.martins@gmail.com (F.R.D.M.M.); mpaiano@uem.br (M.P.); vanessadenardi@hotmail.com (V.D.A.B.); 2 School of Health Sciences, Polytechnic of Leiria, Campus 2, Morro do Lena, Alto do Vieiro, Apartado 4137, 2411-901 Leiria, Portugal; 3 Centre for Innovative Care and Health Technology (ciTechCare), Rua de Santo André-66-68, Campus 5, Polytechnic of Leiria, 2410-541 Leiria, Portugal; 4 Comprehensive Health Research Centre (CHRC), University of Évora, 7000-801 Évora, Portugal

**Keywords:** prison, prisoners, families, COVID-19 pandemic, grounded theory, Brazil

## Abstract

Worldwide, the COVID-19 pandemic represented a health emergency for prisons. This study sought to understand the meanings and experiences through the narratives of prisoners and family members affected by the COVID-19 pandemic in the context of a maximum-security state penitentiary complex in southern Brazil. For this purpose, a qualitative study was developed based on the methodological framework of constructivist grounded theory. Data were collected between February and August 2022 through individual in-depth interviews and field notes. The sample consisted of 41 participants: 28 male prisoners, and 13 family members. Guided by the Charmaz method of grounded theory analysis, the study afforded the core category “Feeling trapped in prison during the COVID-19 pandemic” with three interrelated phases: “Triggering”, “Escalating”, and “Readjustment”. The “Triggering” phase refers to COVID-19-related elements or events that triggered certain reactions, processes, or changes in prison. During the “Escalating” phase, participants became overwhelmed by the suffering caused by incarceration and the pandemic crisis. The “Readjustment” phase involved adapting, reorienting, or reformulating previous approaches or strategies for dealing with a specific situation. Prisons faced complex challenges during the pandemic and were forced to prioritize protecting public health. However, the measures adopted must be carefully evaluated, ensuring their needs and that they are based on scientific evidence. The punitive approach can undermine inmate trust in prison authorities, making it difficult to report symptoms and adhere to preventive measures.

## 1. Introduction

Health threats and inequalities within prisons, especially in overcrowded and under-resourced facilities, are well known [1]. Nonetheless, the COVID-19 pandemic represented a health emergency for prisons globally. Currently, the world prison population is over 11.5 million and, in most cases, prison conditions are unhealthy and precarious [2]. Currently, Brazil has a total prison population of more than 835,000 individuals, including pre-trial/provisional prisoners, which is equivalent to a prison rate of 389 prisoners per 100,000 inhabitants [3]. Of the prison population, 67% of individuals are black and have a low level of education (75%), having never attended high school [4].

From March 2020 to December 2022, there were 115,831 cumulative COVID-19 cases and 703 deaths in prison settings in Brazil [5]. Prisons have notoriously unfavorable conditions—such as precarious physical spaces, scarcity of specialized human resources, lack of adequate healthcare, and the presence of structural violence practices—and concern for the healthcare of prisoner populations has historically been low and fragmented in Brazil [6,7]. The COVID-19 pandemic aggravated the social, economic, and health inequalities in the criminal justice system [8]. From a socioecological perspective, this demands a reflection concerning how individuals are influenced by their environment and the circumstances to which they are exposed.

The socioecological model (SEM) emerged as a multidimensional and multilevel view of the determinants of health, and it played a significant role in the management of the COVID-19 pandemic in prisons [9]. According to this model, human development (including thoughts, emotions, preferences, and behaviors) is determined by multiple influences from the subject’s context. In this sense—and assuming individuals as open systems, constantly affected by circumstances—the prison context acts as a threatening environment capable of enhancing inmate vulnerability. People who experience incarceration are among the most vulnerable members of our communities [10]. Lack of freedom, restrictive living conditions, and poor interaction with other detainees and prison staff have the potential to affect the well-being and mental health status of inmates and leave them more vulnerable [11,12,13] and ‘laid bare as contexts of vulnerability in the pandemic’ [14] (p. 499). Schliehe et al. [15], inspired by Michel Foucault’s biopolitics, used the term “biological sub-citizens”, wherein the prefix “sub” refers to political and economic subordination and the deprivation of health rights, resulting in various forms of marginality and exclusion. While the literature emphasizes vulnerability as a universal condition of human beings [16], other authors concentrate on the social processes that generate vulnerability, as well as the duty of the state and its institutions/systems to decrease its risks and effects [17,18]. Recognizing the complex interaction between the individual and the environment is essential to understanding the reality of the prison population and finding more humane and effective approaches for their social reintegration [19]. This might include implementing evidence-based policies to lessen the negative health effects of the pandemic, such as initiatives to improve mental health and treat mental illness [20,21].

Given that the prison population is already trouble-prone under their “normal conditions” of confinement, one might expect their biopsychosocial needs to deteriorate considerably in times of crisis [22]. Indeed, inmates may suffer greater levels of stress and anxiety because of the risky and unpredictable conditions in which they all live. In addition, professionals and personnel faced extra demands due to the risk of infection to themselves and their families. The fears associated with the COVID-19 pandemic had an impact on inmates and those who had to care for them, as the disease could spread inside prisons [23]. During the months of lockdown, the suspension of visits and the scarcity of activities reduced contact with the outside world and promoted the use of maladaptive coping strategies due to the perception of poor social support [24]. In fact, the impact and implications of the pandemic and restrictions on the lives of prisoners and their families are still unknown [12,25].

At present, most studies have dealt with health issues and the risks associated with the transmission and containment of COVID-19, rather than the pandemic’s effects on those who live in prison settings [26]. Worldwide, few qualitative studies have explored the impact of COVID-19 on prisons [27,28]. Furthermore, to our knowledge, no prior research exists in Brazil using in-depth interviews with people who lived in prisons during the pandemic and their family members.

Grounded in a socioecological perspective, and considering the need for a different look at prisons and the consequences of Brazil’s public health emergency (COVID-19), the research question was the following: based on the narratives of prisoners and families, how was the meaning and experience of incarceration affected by the COVID-19 pandemic? This study aimed to understand the meanings and experiences through the narratives of prisoners and family members affected by the COVID-19 pandemic in the context of a maximum-security state penitentiary complex in southern Brazil.

From an inner perspective, we hope this study will add to the expanding, yet still sparse, body of knowledge on COVID-19’s effects in prisons, including recommendations for further research as well as public health implications.

## 2. Materials and Methods

### 2.1. Study Design

This inductive qualitative study is part of a larger project entitled “Repercussions of COVID-19 on the prison system” that aims to assess the impact of COVID-19 on the Brazilian prison system from the perspective of inmates, families, penitential agents, and health professionals. The present study used the constructivist grounded theory (CGT), a framework that recognizes the subjectivism of reality and the researcher’s involvement in the construction of meanings [29]. This approach aims to understand how individuals interact regarding the phenomenon and focuses on an interpretive understanding of the subjects’ meanings [29,30]. A constructivist researcher “co-constructs experience and meanings with participants” [30] (p. 2). The present study was conducted and reported following the consolidated criteria for reporting qualitative research (COREQ) checklist [31].

### 2.2. Setting, Sample, and Recruitment

The scenario of this study included a large high-security penal establishment, located in the municipality of Maringá, northwest of the State of Paraná, Brazil. This establishment was built to receive pre-trial/provisional male prisoners. However, due to few vacancies in other establishments, it also welcomes convicted persons. At the time of data collection, the unit (with a capacity of 960 people) was occupied by 1100 inmates. The occupancy rate of 115% naturally affects its ability to guarantee adequate accommodation conditions.

Given that daily life in high-security prisons is structured around restrictive policies and measures, these contexts are relevant research sites for assessing the impact of the COVID-19 pandemic on the prison population.

Using a purposive sampling strategy, we recruited two types of voluntary participants: (a) adult inmates (in prison during the pandemic); and (b) adult family members (who visited the prison during the pandemic). Participants were excluded if they had cognitive impairment assessed by the mini-mental state examination [32]. The researchers attempted to include demographic diversity, in terms of age, educational background, and ethnicity.

A member of the prison staff (W.C.B.) supported the research team with the recruitment of participants using the local inmate directory. Potential participants were approached, provided with an information sheet, and given at least 24 h to consider their participation. If participants wanted to participate, an interview appointment was scheduled.

In the current study, sampling began as purposeful and was adjusted to theoretical sampling depending on the data collected. In this sense, data gathering and analysis were simultaneous. Data saturation was achieved with 28 inmates and 13 family members.

### 2.3. Data Collection

Data were collected between June and August 2022. The interviews were only carried out by a female registered nurse (W.C.B.) with clinical activity in prison institutions for over 15 years, and a Ph.D. candidate. A semi-structured interview guide was prepared by the main researcher, based on her professional experience and the available evidence [8,33] and used as an instrument to collect data. Before starting the data collection process, the guide was validated by three experts, all with Ph.D.s, with experience in qualitative research, who validated the content, form, and clarity of the questions, obtaining an agreement of over 90%.

The topic guide was broad, with open-ended questions, allowing in-depth descriptions of meanings and experiences. We asked participants to describe what they understood about the impacts of the COVID-19 pandemic, their experiences, and the barriers and facilitators to overcome the repercussions of COVID-19 in a prison environment. Follow-up questions were asked after each participant’s responses to engage them in a dialogue. At the end of the interview, the participants were asked if they had anything to add.

Due to the introduction of new topics during data collection, the interview guide was adjusted. Thus, as the interviews progressed, the questions were modified to improve comprehension and clarity of the phenomena being examined. As recommended by the CGT, field notes were gathered before, during, and after the interviews, and memos with the researcher’s perspectives were produced to help the study [29].

The interviews were carried out in a specific place in the institution that guaranteed the privacy of the interviews. For security reasons, the door remained open during interviews with inmates, and a penal officer stood outside the room. During data collection, the interviewer chose to wear ordinary clothes instead of a uniform, indicating that data collection would not affect her interaction with the participants and would not interfere with her position in the prison. All interviews were conducted in person. No interviews were repeated. Interviews were audio recorded and completely transcribed, lasting from 25 to 90 min, with an average duration of 40 min.

The quotations were first translated into English and then back into Portuguese to guarantee that they were appropriately translated. Extracts from the interviews were numbered according to the participant’s role, followed by a number indicating the order in which each participant was interviewed, for example, inmates [I-1]; and family members [F-30].

### 2.4. Data Analysis

Descriptive statistics for sample characterization were calculated using frequencies, percentages, minimum, maximum, mean, and standard deviation.

An inductive approach was used to analyze and code the data [29]. Qualitative data analysis was performed using CGT techniques using MaxQDA^®^ software, version 2018 (VERBI Software, Berlin, Germany) [34], as a data management tool.

Following the constructivist perspective, coding involved three stages. Initially, the data were coded inductively, line by line, using a content analysis method. The concepts were identified and grouped into categories and subcategories according to their similarities. In the second stage of coding (focused coding), the developed codes were more specific, selective, and conceptual, as they needed to synthesize and explain broader segments of data. In the last stage (theoretical coding), data analysis was presented with attention to the participants’ main concerns and incorporating theoretical concepts that fit with the data [29,35]. As certain concepts appeared with greater frequency and prominence, subcategories and categories were created that revealed the phenomenon or the central category of the research. Here, the researcher’s perception becomes relevant to identify the central category that represents the most significant process in the research area [29,36].

### 2.5. Study Rigor and Reflexivity

To ensure the accuracy and validity of the research, Charmaz and Thornberg [37] proposed four criteria: credibility, originality, resonance, and usefulness. The interviews were transcribed in detail and the field notes were compared and checked regularly to ensure the credibility of the study.

Credibility was sought through member verification, prolonged engagement, and peer debriefing [29,38]. Member verification took place in seven additional interviews to confirm the emerging theoretical categories. Prolonged involvement in the field allowed participants to feel comfortable with the research team. Peer debriefing with members of the study research team was conducted throughout the research process.

The originality of the findings was established through a comprehensive reflective process that included writing memos, evaluating the existing literature, and using layout techniques in conjunction with the research team [29,36]. The discussion reflects resonance to explain the results within the context of earlier research and suggests applicability to related individuals and circumstances.

An iterative process was conducted to develop the codebook over several meetings with the co-investigators. Hence, to facilitate the emergence of a theoretical model (usefulness criteria), a schematic representation was proposed to inform the phenomenon under study and to illustrate the relationship between the core category and the other categories [29].

Recently, there has been increased interest in the literature about researcher reflexivity, especially in the context of CGT. This interest is particularly relevant in constructivist approaches since research is understood as a co-constructive process [29]. This research was enriched through reflexivity in terms of transparency and credibility [39]. This allowed for more careful and critical consideration of the impact of the researchers’ perspectives and influences on the development of this study. The research team included a nurse (W.C.B.) with experience in caring for the prison population. The remaining team members had experience in qualitative research and were affiliated with the constructivist epistemological perspective (M.A.S., L.C., A.M.G., F.R.M.M., M.P., V.D.A.B., and C.L.).

### 2.6. Ethical Issues

The research received approval from the State University of Maringá—UEM Research Ethics Committee (approval number 3.211.746) and complied with all the guidelines outlined in the Declaration of Helsinki. Before each interview, informed consent—which included permission for audio recording—was obtained in writing. The option to withdraw from the study at any moment was made clear to participants. There was no monetary reward for participating. Nursing support to participants was provided upon request, considering physical and psychological discomfort during interviews.

## 3. Results

### 3.1. Sample Characteristics

The sample included 41 participants, of whom 28 were inmates, with an average age of 36 years and an average time of imprisonment of around 27 months. Participants were mostly of African descent (47%), with less than eight years of schooling (54%), and with children (75%). The most prevalent crime was drug trafficking (46%), and the criminal recidivism rate was around 79%. Around 57% of the inmates worked inside the prison, and many were infected with SARS-CoV-2 (43%).

The second sample group was composed of 13 family members, only women, with a mean age of 40 years. Most were white/Caucasian (62%), with nine or more years of study (62%). Most had children (85%) and were employed (54%). Table 1 depicts the sample description.

### 3.2. Overview Theory

**Figure 1:**
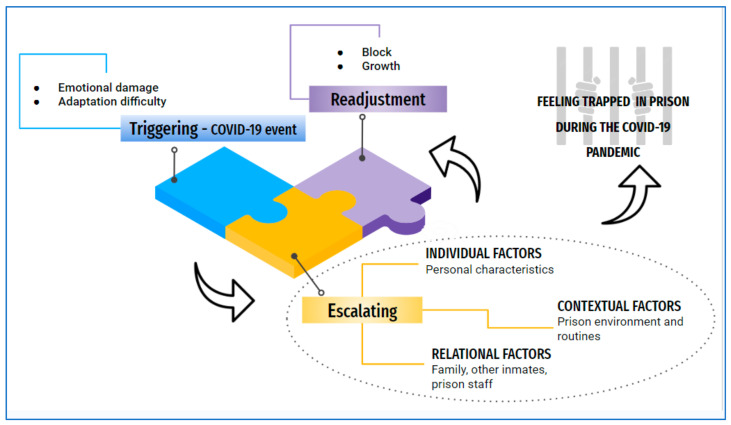
Overview theory—meanings and experiences of prisoners and family members affected by the COVID-19 pandemic.

The categories and subcategories were related through an analytical process formed by three phases: triggering, escalating, and readjusting. Afterwards, interactions between categories were established around a central category “feeling trapped in prison” (see Figure 1). The prison context is characterized by restricting the freedom of individuals who have committed crimes and been convicted. This restriction implies the deprivation of multiple rights and the limitation of contact with the outside world. The COVID-19 pandemic brought about significant changes that further affected this already existing feeling of seclusion.

#### 3.2.1. Triggering Phase: COVID-19 Event

The triggering phase reflects that during the COVID-19 pandemic, inmates were highly susceptible to emotional damage, which hindered their adaptation to the new reality. Prisoners showed different concerns regarding the pandemic, associated with fear, the transmissibility of the virus, and the lethality of the disease. Participants shared:

*I was afraid because it was a disease that was killing a lot of people at the time.*

(I-11)

*The virus is transmitted through saliva and the air itself. I got infected by sharing food with an infected person… then, I ate what he had already bitten, and I caught it.*

(I-16)

The fear of death was evidenced in the speech of many participants, mainly related to the association of comorbidities with contracting the disease. In this regard, I-3 mentioned:

*Besides, I have bronchitis and rhinitis, which would be worse for me… because if I caught this disease, I don’t know if I would survive.*

The media, such as television, were fundamental in providing information about the COVID-19 pandemic. For some inmates, television news was the only medium they followed, even identifying the possibility of fake news. In this sense, the fear and anxiety associated with the disease and the possibility of superficial and unsupported information contributed to aggravating the social stigma against infected people, groups of people, or affected places, but also against people who left a situation of isolation or quarantine, even when they no longer posed a risk of spreading the virus. Most participants referred to the disease without naming it, either because of fear of the unknown or a lack of knowledge and understanding.

*I don’t know anything about the disease, what I hear on TV and we hear a lot of fake news. So, we have no way to guide ourselves. I know that it (the disease) kills people, I know that it is transmitted. I feel that sometimes some discrimination is generated against those who are infected by the virus.*

(I-12)

*What I see on television is that it is a virus that can kill, it is transmissible, it attacks the lungs and, in some cases, it brings sequelae. That’s what I know about it (the disease).*

(I-9)

Likewise, the prisoners’ relatives expressed their fears of contracting the disease and their concerns for the health of their loved ones, even manifesting fears about the possibility of death. Additionally, they highlighted the precarious structure of prison units, highlighting the lack of hygiene materials and food restrictions as sources of concern.

*… if there were no conditions out there, I kept imagining in here, it was very worrying… I was out there and worried about him, if he got contaminated and he didn’t eat properly, in prison food was very precarious.*

(F-5)

*I felt horrible, I thought about everything because at home we protect each other… and here how did he protect himself? Because there were no masks or alcohol… my fear was that he would get caught and the worst would happen. I was worried about him catching COVID-19, having low immunity, and perhaps dying, all that was going through my head.*

(F-11)

#### 3.2.2. Escalating Phase: Balancing between Individual, Relational, and Contextual Spheres

During the escalating phase, participants become overwhelmed by the suffering caused by incarceration and the pandemic crisis. Under regular circumstances, the prison system is recognized as a hostile and difficult environment. With the pandemic, inmates had to deal with an even greater level of suffering than merely being in the prison system. Several intervening factors contributed to facilitating or challenging the escalating phase during the new pandemic reality. 

Some factors were **individual**, namely, several specific characteristics of this population, including pre-existing vulnerabilities, such as mental or physical health problems, which worsened during the COVID-19 pandemic. Prisoners were even more likely to suffer emotionally from isolation, poor prison conditions, and the possibility of being infected with the SARS-CoV-2 virus.

*I have a chronic disease, and colleagues said, if you have to get it, it’s you… because I have diabetes, arrhythmia, and blood pressure problems, I was the one with the highest risk of getting infected and developing a serious disease.*

(I-4)

Restrictions due to containment measures against infection—such as the suspension of work, educational and religious activities, and visits to the prison environment—also contributed to emotional instability. This highlighted the need for resilience and psychological flexibility as adaptive strategies in the face of adversity. As stated,

*If the person does not have a firm mind, they do something stupid, they go crazy…*

(I-17)

*For as long as I can remember, I have never been in this type of situation, it was paralyzing, so many rules were imposed, and it was a very difficult situation. Only by having a good capacity for adaptation is it possible to deal with this situation.*

(I-10)

However, several inmates, for fear of being isolated, omitted their real health status and did not request support from the prison’s health services. Avoidance as a coping strategy seems to have provided temporary relief from stressors.

*We didn’t ask for care because everyone was afraid of going to Campo Mourão (isolation unit), and we “preferred to suffer” because if we asked for medicine, someone would be suspicious.*

(I-9)

*I got really bad, with a headache, and a stuffy nose, it was one of the worst colds I’ve ever had. I had a fever, and I didn’t tell anyone, people were isolated, so I kept quiet, and I didn’t ask for care.*

(I-21)

**Relational factors** are linked to support networks and economic resources. Prisoners and family members portrayed feelings of emotional distress because the infection and its control measures could threaten family ties. One participant said:

*I was very afraid of the disease, it was very worrying, I suffered because of the fear of him dying or me dying and we not seeing each other anymore.*

(F-13)

On the other hand, an inmate adds:

*I feared not being able to give the last hug to my son, to my family.*

(I-11)

Negative experiences related to the suppression of visits were underlined by both inmates and family members, who reported homesickness, fear of abandonment, and non-compliance with the parental role.

*The main difficulty was mostly longing for the family, two years without being able to see them.*

(I-11)

*… My four-year-old son is autistic. When my husband was arrested, he called him every night (he cried), and that went away, and he forgot about his father.*

(F-4)

*… my daughter was born, and I was here… I was apprehensive to know how she was doing. Today she is two years old. It was tense. I accompanied the other two children, I supported them, I’m a good father, and now I can’t be there, and that worries me.*

(I-19)

*We men have some paranoia and fear that the woman will leave us. We keep that in mind. Men are complicated, they are jealous, and afraid of being exchanged. Will she be able to stand this long without me? She tells me to stop being silly; we have a life together, after all. What is important is for me to stop making mistakes.*

(I-23)

One of the participants spoke of a kind of social death that results from the impossibility of having contact with family members, using the following metaphor:

*… being here without contact with the family is the same thing as being buried alive.*

(I-3)

While separation and absence from your loved one can be difficult to deal with, some inmates do not want to be a burden on the family. In this regard, I-26 said:

*… I ask my family not to send money. I don’t want my family to keep paying the price because I’m wrong… so I don’t ask them for anything.*

On the other hand, daily living with cellmates strengthens the bond between inmates and influences the positive assessment of being isolated. The relational tension between inmates and prison staff was also mentioned, given the fear of the latter transmitting the infection from the community into the prison.

*When I was isolated due to the infection, I went to Campo Mourão (isolation unit), there were six of us, three in each cell, and we all got along well… it was 14 days. Where we were, there was a small hole in the wall and that helped us talking between cells.*

(I-11)

*There was a belief that criminal-police officers were potential transmitters of COVID-19 because they were in transit between the prison and the community, including delivering food.*

(I-7)

*We have no contact with anyone other than the employees, you know? Even though they were wearing a mask, sometimes they brought our food. So, if the virus is to be passed on, it is because they are infected…*

(I-17)

The **contextual domain** includes factors such as the lack of sanitary conditions in prison, providing information about the disease, and changing routines and habits experienced or imposed by restrictions. Many inmates struggled with limited access to hygiene materials and personal protective equipment. Additionally, physical distancing was difficult to ensure, and access to healthcare was limited.

*There were eight of us in a cell. We were afraid of transmitting the disease. There was no soap for bathing or toothpaste. I got tired of brushing my teeth with soap. There was no liquid soap for washing clothes. There was a lack of hygiene products.*

(I-21)

*We didn’t have gel alcohol; it was only in some spaces, like at the entrance to the courtyard and the entrance to the health sector. In the cells, we didn’t have any! As for hygiene products, my wife sent them, and the same happened with the masks that have to come from home, where they have them, but there are very few of them…*

(I-12)

Several participants highlighted other limitations, including overcrowding, and excessive and abusive confinement leading to serious human rights violations.

*… he explains to me that the cell is small and there are eight people, a shower nearby, a toilet hole nearby; it was as if we lived inside the bathroom, and there were no conditions.*

(F-1)

*In a cell, two sleep on the floor and six on beds, there are no conditions.*

(I-11)

*It was desperate. They put me in an isolation cell with six beds… pitch black, it was worse than the other cells. There were prisoners coughing, and I thought I was going to die!*

(I-14)

At the same time, the “condition of being imprisoned” resulted in a constant threat to the human dignity of prisoners and their families. Several participants reported communication difficulties with prison officers; in particular, I-12 stated:

*… I remember when I arrived, the guards said ‘Another who has come to die’, in a clear allusion to the stereotyped view that permeates contexts where vulnerability is more apparent.*

Other participants add:

*… the prison system leaves much to be desired in this regard (human dignity). They give a lot to the staff; if it is a criminal, the person does not deserve care.*

(I-22)

*(…) We are paying a penalty, that’s right, it has to be like that. But there are people (penal agents) who come here to be vigilantes, hurting, mistreating, and saying that you are this and that…*

(I-23)

*I am not to blame for the wrong things he has done. I never agreed with anything he did wrong, but it seems that the family also has to pay for his mistake… I feel really bad about that.*

(F-13)

*When I go through the metal detector machine, I get nervous, they tell me to go back and go through it again… The other time I came, they said I was bringing something. A person feels humiliated. I wanted to cry, and I said “How come I have something? I just came to visit my son”.*

(F-7)

Participants recognized that the measures imposed by the institution were necessary to mitigate infection in the prison system, highlighting the suspension of face-to-face visits. However, this suspension was not accompanied by alternative communication strategies, and those that did exist were limited.

*… it was important, because if not, everyone would have died in here; we don’t understand that it was good. Not only for us, but for our family as well, because, if not, our family would have to be all crowded and together, because there is no structure to separate and receive everyone, and outside it is very crowded.*

(I-9)

*Regarding the restrictions on visits, I think it was necessary because the world stopped… It wasn’t going to be the penitentiary system that was going to be opened.*

(F-10)

*There was a letter that took two months to arrive. Many prisoners started to use letters more during the pandemic.*

(I-21)

*Not being able to visit him was distressing. We had no contact, and no news. It took a long time because it took everyone by surprise and the letters took a long time to arrive.*

(F-5)

Over time, there was a delay in returning to face-to-face visits, as there was an easing of restrictive measures across the country but accompanied by discrepant guidelines between the different Brazilian states.

*When the measures eased outside, they could have eased in here, too, because it took a long time, because outside everyone was already going to places. It was possible to go without a mask, and, for us, it took all this time.*

(I-3)

According to the contingency plan adopted by the penal unit, food that was previously brought by family members was now delivered by a courier (Sedex), and food often arrived in unsuitable conditions for consumption, which negatively affected the finances of relatives.

*Sedex took 20 days to deliver and when it arrived, everything was spoiled.*

(I-5)

*… I’ve already gotten rotten bread, you know? It spoiled a lot of things.*

(I-17)

*Getting there is enough, but the difficult thing is not for us but for our family; it is expensive to send. It gets harder. If I could go back to normal, it would be better.*

(I-13)

*Ah… it was difficult because things are not cheap… I had to pack.*

(F-7)

#### 3.2.3. Readjustment Phase: Between Block and Growth

The pandemic context brought significant and challenging changes for everyone, demanding a capacity to readjust. Some participants were able to adapt and accept the new reality, finding ways to grow and deal with difficulties. However, others faced difficulties in readjusting, becoming “stuck” in the present, with no future prospects.

For many prisoners, working inside prison provided a sense of personal worth, and enabled them to prepare for life outside prison after serving their sentences.

*It’s really good to work; when you’re locked up, you only think about bad things, and the people who are beside you don’t add anything. And, working, you are focused on something, you are motivated by something, and you talk to other people who can add good things to your life.*

(I-28)

*I thank God every day for being here in the garden; it doesn’t even feel like a prison, there’s a bit of freedom. I do what I like, which is cutting hair, and I have many clients. I try to do a good job…*

(I-28)

*For the mind, working helps a lot.*

(I-21)

Concomitantly, work activity in the prison environment contributed to the proper functioning of the unit and brought benefits to the inmates, such as sentence remission, pecuniary gains, and professional training. In this context, one participant reported that working favored social reintegration and offered skills when leaving prison:

*… before I was arrested, I worked with truck mechanics. At the time, I thought I couldn’t charge people. I didn’t know that. I’m learning now; they’re teaching me how to charge and handle money. I had no idea about that. Now, I am better prepared for when I leave (prison).*

(I-24)

One of the prison’s strategies for inmates to contact their families was virtual visits (online). Although for some participants the experience was positive, others experienced difficulties with the technology, due to a lack of digital literacy.

*It’s bad. I was in agony, and I only had news of the family when I went to the videoconference, when I had the virtual visit. That moment was very good; it helped.*

(I-8)

*I feel happy. Even if I was far away, at least I was seeing; I was fine, and it gave me relief, even if it was through the screen.*

(F-12)

*For me, it didn’t help at all. It was once a month, and there were times when other prisoners were talking on the side; it was not possible to hear anything the family was saying, the system blocked it. The image disappeared, and then it came back. As there were other prisoners in the room, I couldn’t hear what the family was saying, so I couldn’t understand anything.*

(I-13)

*At first, I thought it was a little strange, but then I got used to it and it was good. I was sad when I hung up. It was very fast… having news made me feel calmer.*

(F-6)

Although seclusion has a punitive connotation, many inmates transformed seclusion into a life lesson, developing a new perspective of the world, hitherto unknown.

*I can’t wait to get out of here. I don’t want this life for myself anymore. This is enough! This place is too much—jail is enough for me, I’ll get a job.*

(I-8)

*It helped to increase my faith, to do things that I wouldn’t stop to do on the street; to appreciate things, too, which sometimes we don’t appreciate on the street, and to learn to value the simplest things. With this pandemic, I don’t know what could happen; at any moment a family member might lose something. It served to increase my faith.*

(I-11)

*I believe I’m going to leave here as a different person. I already have a very focused mindset on what I’m going to do out there, it’s already well-planned.*

(I-26)

*I made mistakes, but I don’t want to anymore. I want from now on to be a better man and person, living with agents is very good for us, and for me and my family it has been a blessing.*

(I-24)

In parallel, inmates underlined that, although the health service’s attendance was positive, it was necessary to invest in more effective communication and information circuits in accessing said services.

*All the consultations I’ve had here until today, I don’t question them, I was attended to, they gave me attention. At least for me, it was resolving.*

(I-17)

*You should talk to all the on-call staff and all the employees who work in the galleries, in general, all the employees, and explain the importance of this medical assistance for the detainees so that they can open their minds and have more dialogue.*

(I-23)

*It would be good if the unit had better organization. The guards needed to organize themselves to see who is most in need. Because if you don’t have to do what I did, scream, to get care.*

(I-19)

Finally, some of the participants presented a fatalistic discourse and positioned themselves in a position of subservience within the prison system and of resigned acceptance of the reality they were experiencing, limiting their ability to readjust.

*I know that this place here will never change, because we’re stuck, and the government doesn’t even care about us.*

(I-2)

*In part, we even understand, but in other parts, we don’t… because, like it or not, we are already trapped. So, we depend on them for everything—to eat, to drink, for everything. So, there are times when we don’t care the way it should be.*

(I-17)

## 4. Discussion

This qualitative study sought to understand the meanings and experiences through the narratives of prisoners and family members affected by the COVID-19 pandemic. Over time, the pandemic scenario changed life in prison and affected the social identity of individuals, their structuring markers, and their biographical continuity. In this regard, our findings identified participants’ lack of knowledge and fear of the disease. Other studies report similar results, indicating that fear, psychological impact, and uncertainty associated with COVID-19 were prevalent in prison populations [12,13,28,40]. In this sense, it is necessary to define appropriate guidelines for the psychological needs of inmates and to improve information and communication between inmates and prison staff [13,25,41,42,43]. Obtaining information about COVID-19 might help people feel less anxious or depressed [44], but a lack of knowledge can make inmates feel more depressed [45]. It is easy to imagine how something unknown, such as COVID-19, might be represented in a distorted or exaggerated way. This observation becomes particularly relevant due to the low educational levels of the prison population and the limited access to information in prison, which can exacerbate misinformation and fear [13,42,46,47].

The COVID-19 pandemic affected everyone’s lives, but, for individuals who were already in a situation of vulnerability, the crisis had a more significant impact [25]. The lack of adequate emotional support and the stigma associated with the prison system made the situation difficult for this population and their families, making the readjustment process even more difficult [12].

Participants also reported doubts about the care they would receive in the event of an infection, which demonstrates a lack of confidence in the prison system’s ability to contain the disease or provide adequate medical treatment [41,43]. The available evidence points to a lack of health coverage in the prison system, as demonstrated by the lack of testing campaigns and adequate treatment strategies to contain the escalation of the disease [48].

Furthermore, the reorganization of services in the prison environment resulted in negative experiences for prisoners and their families, due to the physical distance imposed by the restrictive measures. The connection of incarcerated people with their families is essential to strengthening family relationships, in addition to helping the safe transition to the community after release [49,50]. Notably, family ties protect individuals from mental health problems. This factor was among the most highlighted by participants in the current study. In this regard, Tadros [49] states that relational therapy is a therapeutic approach that can be useful to help detainees and their families deal with social isolation and anxiety during the COVID-19 pandemic, promoting emotional connection and resilience.

The lack of clear information about the pandemic intensified relatives’ uncertainty and insecurity regarding the conditions experienced within the walls [42,51]. These concerns, together with the challenges inherent to the prison environment, created a scenario of accentuated complexity and demanded effective actions by competent authorities [42].

Studies on the impact of COVID-19 from the perspective of family members are scarce [51,52]. Our findings showed that one of the main challenges indicated by family members was the lack of prison visits, which caused great concern over health and physical and mental well-being. Many reported the harrowing and desperate experience of being unable to visit and feared inmate exposure to unhealthy living conditions in prison [51]. Furthermore, lack of communication and contact can be extremely difficult for families, who are already dealing with the emotional stress of having a loved one in prison [52]. Some family members understand the need for measures to prevent the spread of the virus, restricting contact to letters and virtual visits. In parallel, they sought other forms of supporting their family members in prison, sending groceries and other items necessary for their hygiene [51].

However, the COVID-19 pandemic has brought financial challenges to family members, who face difficulties in sending groceries and personal hygiene items needed by inmates [51]. Many families already faced precarious financial situations, even before the pandemic, and the health crisis only worsened the situation [51]. With the suspension of prison visits, family members were no longer able to personally bring the necessary supplies and items, adding shipment costs to their financial burden [51,53]. This aspect generated concern and anguish, as many prisoners depended on their families to ensure adequate food and personal hygiene [54]. Prison authorities must consider this reality and seek alternatives to ensure that prisoners have access to groceries and personal hygiene items, without further financial burden for their family members.

When an individual is arrested, the punishment is often tacitly shared with his family, which are often part of social groups marginalized by society and the State itself [54]. Thus, in addition to the emotional distress caused by separation from a loved one, prisoners’ families also face a variety of obstacles and social stigma. Often, they have to deal with financial and emotional difficulties, and are seen as accomplices or responsible for the crimes committed by their imprisoned family members. This reality is a reflection of the social exclusion and inequality that affects a large part of the Brazilian population [54].

This reality of the prisoners’ families is directly related to COVID-19, and was identified by Mezzina et al. [55] as a “syndemic”—meaning that the consequences of the disease are compounded by social and economic disparity. These disparities are magnified by human rights violations, stigma, and discrimination. The pandemic particularly affects vulnerable and marginalized groups, who face structural discrimination and violence, being considered a “disease of inequality” as it negatively and disproportionately affects the most socially and economically disadvantaged [55]. In this sense, it is crucial to increase the options for activities inside prison and expand electronic communications, allowing prisoners to communicate both with their families and with health professionals. The prison system must recognize and promote effective communication between detainees and their families, expanding opportunities in this regard and facilitating interaction with health professionals, particularly through telematics [13].

Our findings indicate that prison overcrowding is an important factor in the spread of the virus. Studies suggest that compassionate liberation as a depopulation measure [1,56] is capable of reducing the phenomenon. However, despite the possibility of reducing overcrowding in prisons, there is a risk of releasing prisoners without housing conditions and, thus, increasing crime rates. Other effective measures used a combination of education, screening, testing, isolation, quarantine, physical distancing, vaccination, and sanitization [1,57]. Political efforts must consider extrication as an effective strategy to reduce the prison population while promoting greater investment in communities affected by mass incarceration [56].

The mass release of people into the community can create difficulties for these individuals, and it is necessary to guarantee adequate protections, such as decent housing and access to health services for those who return to the community after serving their sentences. To ensure a satisfactory and effective transition, measures must be underpinned by strong leadership and collaborative work between prison systems, non-governmental organizations, and health and social care partners [13,56].

Our research also found that some inmates recognized that the measures adopted by the prison system were adequate and necessary to face the pandemic, despite their repercussions on individual and relational well-being. Similar results were found in Pyrooz’s [43] study, in which prisoners considered the institutional response positively, alleviating their concerns about COVID-19, although they expressed concern about its maintenance over time.

Our findings also highlight that medical isolation or confinement during the COVID-19 pandemic generated reluctance among inmates to report symptoms. This can impair the effective control of the infection, corroborating other studies that also highlight this concern [41,42]. This reluctance to report symptoms, for fear of being subjected to conditions similar to disciplinary and/or administrative solitary confinement, should not be overlooked [42]. On this point, Cloud et al. [58] (p. 2738) say that “it is essential to clarify the critical differences between punitive solitary confinement and the ethical and humane use of medical isolation and quarantine during a pandemic”. 

The adverse impacts of solitary confinement are widely documented due to its negative psychological, neurological, and physiological effects [59]. In addition, it is important to provide additional mental health support, address substance use, and ensure the provision of physical care for those in medical isolation in order to strike an appropriate balance between individual harm and public health goals.

### 4.1. Strengths and Study Limitations

We chose the grounded theory approach in this study because it provides empirical knowledge when prior hypotheses are non-existent. This methodology made it possible to explore and develop theories and concepts from the data collected, rather than from pre-established assumptions. In this way, the research questions were approached in an open and exploratory way, allowing new insights and understandings to emerge from the data. Qualitative research is a valuable resource for exploring the complex dynamics of prison life during the context of the COVID-19 pandemic.

Despite its strengths, some limitations were identified. First, accessing prison populations for research purposes is a complex challenge, especially when dealing with maximum-security prisoners. Second, collecting data in prisons during a pandemic implies even greater logistical and practical difficulties related to security and healthcare, since prison populations are highly susceptible to the spread of contagious diseases. Third, due to the small sample size and qualitative approach of this research, its generalizability is constrained. Future research using quantitative techniques may be useful for compiling more thorough data on the impact of COVID-19 in prisons. In addition, the study did not distinguish between people with various medical needs and various health problems, nor did it distinguish between inmates according to the crime they committed or the length of their sentence. Depending on their health state and length of incarceration, convicts may manage their health in various ways. Future studies that take these factors into account are necessary. The viewpoints of male convicts were also a focus of this investigation. Future research should thus focus on the experiences of women in prison, since they may vary greatly from those of male inmates.

Finally, data for this research were gathered two years after the COVID-19 pandemic’s onset. Undoubtedly, this crisis presented serious difficulties for prisoners and their families, and revealed weaknesses in the prison system. Further studies may revise the conclusions of the current study to reflect adjustments made to prison practices as a result of the pandemic.

### 4.2. Implications for Practice

There has been a dearth of studies on inmate populations because of the challenges of research in prisons [60,61], as well as the view of inmates as a “vulnerable” population. Our study has discovered that, in addition to advancing clinical and scientific knowledge, research involving such a group may also directly benefit the participating inmates. Participants specifically mentioned feeling informed or enlightened as a consequence of participating in the study. It is important to attend to the needs of inmates and the factors that affect them. The viewpoint of prisoners and their capacity to influence their own health were disregarded in this situation.

The findings of this study also have important implications for prison staff and how they should be prepared to deal with the various impacts of the pandemic and offer adequate support to incarcerated people. This includes providing comprehensive healthcare, promoting recreational and occupational activities, providing emotional and psychosocial support, and ensuring access to resources and services that can assist with social reintegration after incarceration.

For decision-makers and stakeholders, the results of this study highlight the need to adopt comprehensive measures to deal with the effects of the pandemic on incarcerated people. This includes ensuring access to adequate healthcare, protecting human rights, and promoting measures aimed at re-socializing and reintegrating these people into society. Policies should be sensitive to the particularities of this population, one deprived of freedom, and consider their physical, mental, and social health needs [62].

Finally, hearing the participants’ stories in their own words may make it easier for authorities to understand the specific meanings this understudied group of people assigns to life in jail. To prepare for future pandemic events, tailored psycho–educational and supportive interventions should be developed, using the many ways that inmates and families describe their experiences [63]. Our findings could also assist in implementing evidence-based strategies to facilitate the adaptation process and emotional resilience during the recovery phase.

## 5. Conclusions

The findings of this research show the significant impact of the COVID-19 pandemic on people deprived of liberty and their families, triggering a highly challenging process characterized by “feeling trapped in prison”. Some measures adopted to mitigate the spread of the virus were identified as questionable and punitive, and could even encourage unfavorable reactions, such as resistance to reporting symptoms. However, many recognize that these measures were necessary to control infection within prison units. The punitive approach can undermine inmates’ trust in prison authorities, making it difficult to report symptoms and adhere to preventive measures. In addition, it can intensify the feeling of confinement and increase the emotional tension of the detainees.

## Figures and Tables

**Table 1:** Sample description.

Variables	Inmates (*n* = 28)	Family Members (*n* = 13)
**Age (years)**	36 ± 11.12 (23–67)	40.07 ± 10.28 (25–60)
Mean ± SD (range)		
**Sex**		
Male	28 (100)	0
Female	0	13 (100)
**Ethnicity**		
White/Caucasian	12 (42.8)	8 (61.5)
African descent	16 (57.2)	5 (38.5)
**Education**		
≤8 years	15 (53.5)	5 (38.5)
≥9 years	13 (46.5)	8 (61.5)
**Have Children**		
Yes	21 (75.0)	11 (84.6)
No	7 (25.0)	2 (15.4)
**Work activity**		
Yes	12 (42.8)	7 (53.8)
No	16 (57.2)	6 (46.2)
**Imprisonment time (months)** mean ± SD (range)	27.30 ± 14.24 (13–60)	N/A
**Criminal recidivism**		
Yes	22 (78.6)	N/A
No	6 (21.4)	
**Reason for arrest**		N/A
Assault/theft	9 (32.2)	
Crimes against life	6 (21.4)	
Drug trafficking	13 (46.4)	
**History/presence of COVID-19**		
Yes	12 (42.8)	8 (61.53)
No	16 (57.2)	5 (38.47)

N/A—Not applicable.

## Data Availability

All data generated or analysed during this study are included in this article. This article is based on the first author’s doctoral thesis in Nursing at the State University of Maringá—Brazil.

## References

[1] Esposito M., Salerno M., Di Nunno N., Ministeri F., Liberto A., Sessa F. (2022). The Risk of COVID-19 Infection in Prisons and Prevention Strategies: A Systematic Review and a New Strategic Protocol of Prevention. Healthcare.

[2] Fair H., Walmsley R. World Prison Brief, 3rd ed.; Institute for Crime & Justice Policy Research. https://www.prisonstudies.org/sites/default/files/resources/downloads/world_prison_population_list_13th_edition.pdf.

[3] World Prison Brief: Brazil Institute for Crime & Justice Policy Research. https://www.prisonstudies.org/country/brazil.

[4] Bartos M.S.H. (2023). National Comprehensive Health Care Policy for People Deprived of Liberty in the Prison System: A reflection from the per-spective of intersectorality. Cien Saúde Colet..

[5] Conselho Nacional De Justiça Boletim do COVID-19 No Sistema Prisional. https://www.cnj.jus.br/wp-content/uploads/2023/01/boletim-covid-19-dezembro2022.pdf.

[6] Neto F.J., Miranda R.B., Coelho R.d.A., Gonçalves C.P., Zandonade E., Miranda A.E. (2019). Health morbidity in Brazilian prisons: A time trends study from national databases. BMJ Open.

[7] de Vasconcelos N.P., Germain S., Yong A. (2023). Business as usual: Inequality and health litigation during the COVID-19 pandemic in Brazil. Beyond the Virus.

[8] Murdoch D.J. (2020). British Columbia Provincial Corrections’ Response to the COVID-19 Pandemic: A Case Study of Correctional Policy and Practice. Vict. Offenders.

[9] Turnock B.J. (2015). Public Health: What It Is and How It Works.

[10] Kinner S.A., Young J.T. (2018). Understanding and Improving the Health of People Who Experience Incarceration: An Overview and Synthesis. Epidemiol. Rev..

[11] Pękala-Wojciechowska A., Kacprzak A., Pękala K., Chomczyńska M., Chomczyński P., Marczak M., Kozłowski R., Timler D., Lipert A., Ogonowska A. (2021). Mental and Physical Health Problems as Conditions of Ex-Prisoner Re-Entry. Int. J. Environ. Res. Public Health.

[12] Mendes R., Baccon W.C., Laranjeira C. (2023). Fear of COVID-19, Mental Health and Resilient Coping in Young Adult Male Inmates: A Portuguese Cross-Sectional Study. Int. J. Environ. Res. Public Health.

[13] Johnson L., Gutridge K., Parkes J., Roy A., Plugge E. (2021). Scoping review of mental health in prisons through the COVID-19 pandemic. BMJ Open.

[14] Sparke M., Anguelov D. (2020). Contextualising coronavirus geographically. Trans. Inst. Br. Geogr..

[15] Schliehe A., Philo C., Carlin B., Fallon C., Penna G. (2022). Lockdown under lockdown? Pandemic, the carceral and COVID-19 in British prisons. Trans. Inst. Br. Geogr..

[16] Herring J. (2016). Vulnerable Adults and the Law.

[17] Fineman M. (2010). The vulnerable subject and the responsive state. Emory Law J..

[18] Laranjeira C., Piaça I., Vinagre H., Vaz A.R., Ferreira S., Cordeiro L., Querido A. (2022). Vulnerability through the Eyes of People Attended by a Portuguese Community-Based Association: A Thematic Analysis. Healthcare.

[19] Otugo O., Wages B. (2020). COVID-19: The Additional Sentence for the Incarcerated. Health Equity.

[20] Kim H., Hughes E., Cavanagh A., Norris E., Gao A., Bondy S.J., McLeod K.E., Kanagalingam T., Kouyoumdjian F.G. (2022). The health impacts of the COVID-19 pandemic on adults who experience imprisonment globally: A mixed methods systematic review. PLoS ONE.

[21] Hewson T., Shepherd A., Hard J., Shaw J. (2020). Effects of the COVID-19 pandemic on the mental health of prisoners. Lancet Psychiatry.

[22] Liebrenz M., Bhugra D., Buadze A., Schleifer R. (2020). Caring for persons in detention suffering with mental illness during the Covid-19 outbreak. Forensic Sci. Int. Mind Law.

[23] Kothari R., Forrester A., Greenberg N., Sarkissian D., Tracy D. (2020). COVID-19 and prisons: Providing mental health care for people in prison, minimizing moral injury and psychological distress in mental health staff. Med. Sci. Law.

[24] Hewson T., Green R., Shepherd A., Hard J., Shaw J. (2020). The effects of COVID-19 on self-harm in UK prisons. BJPsych Bull..

[25] Sánchez A., Simas L., Diuana V., Larouze B. (2020). COVID-19 in prisons: An impossible challenge for public health?. Cad. Saúde Pública.

[26] Maycock M. (2022). ‘Covid-19 has caused a dramatic change to prison life’. Analysing the impacts of the Covid-19 pandemic on the pains of imprisonment in the Scottish Prison Estate. Br. J. Criminol..

[27] Wainwright L., Senker S., Canvin K., Sheard L. (2023). “It was really poor prior to the pandemic. It got really bad after”: A qualitative study of the impact of COVID-19 on prison healthcare in England. Health Justice.

[28] Suhomlinova O., Ayres T.C., Tonkin M.J., O’reilly M., Wertans E., O’shea S.C. (2021). Locked up While Locked Down: Prisoners’ Experiences of the COVID-19 Pandemic. Br. J. Criminol..

[29] Charmaz K. (2014). Constructing Ground Theory: A Pratical Guide through Qualitative Analysis.

[30] Tie Y.C., Birks M., Francis K. (2019). Grounded theory research: A design framework for novice researchers. SAGE Open Med..

[31] Tong A., Sainsbury P., Craig J. (2007). Consolidated criteria for reporting qualitative research (COREQ): A 32-item checklist for interviews and focus groups. Int. J. Qual. Health Care.

[32] de Melo D.M., Barbosa A.J.G. (2015). O uso do Mini-Exame do Estado Mental em pesquisas com idosos no Brasil: Uma revisão sistemática. Cienc. Saúde Coletiva.

[33] Comartin E.B., Victor G., Ray B., Nelson V., Whitehead T., Kubiak S. (2022). County jails’ responses to COVID-19: Practices, procedures, and provisions of behavioral health services. Psychol. Serv..

[34] Rädiker S., Kuckartz U. (2019). Analyse Qualitativer Daten mit MAXQDA.

[35] Lindqvist H., Forsberg C. (2022). Constructivist grounded theory and educational research: Constructing theories about teachers’ work when analysing relationships between codes. Int. J. Res. Method Educ..

[36] dos Santos J.L.G., da Cunha K.S., Adamy E.K., Backes M.T.S., Leite J.L., de Sousa F.G.M. (2018). Data analysis: Comparison between the different methodological perspectives of the Grounded Theory. Rev. Esc. Enferm. USP.

[37] Charmaz K., Thornberg R. (2021). The pursuit of quality in grounded theory. Qual. Res. Psychol..

[38] Padgett D.K. (2017). Qualitative Methods in Social Work Research.

[39] Gentles S., Jack S., Nicholas D., McKibbon K. (2014). Critical Approach to Reflexivity in Grounded Theory. Qual. Rep..

[40] Crowley D., Cullen W., O’Donnell P., Van Hout M.C. (2020). Prison and opportunities for the management of COVID-19. BJGP Open.

[41] Liu Y.E., LeBoa C., Rodriguez M., Sherif B., Trinidad C., del Rosario M., Allen S., Clifford C., Redding J., Chen W.-T. (2022). COVID-19 Preventive Measures in Northern California Jails: Perceived Deficiencies, Barriers, and Unintended Harms. Front. Public Health.

[42] Song M., Kramer C.T., Sufrin C.B., Eber G.B., Rubenstein L.S., Beyrer C., Saloner B. (2023). “It was like you were being literally punished for getting sick”: Formerly incarcerated people’s perspectives on liberty restrictions during COVID-19. AJOB Empir. Bioeth..

[43] Pyrooz D.C., Labrecque R.M., Tostlebe J.J., Useem B. (2020). Views on COVID-19 from Inside Prison: Perspectives of High-security Prisoners. Justice Eval. J..

[44] World Health Organization (2020). Preparedness, Prevention and Control of COVID-19 in Prisons and Other Places of Detention.

[45] Okoro J., Odionye T., Nweze B., Onuoha M., Ezeonwuka C., Owoh J., Nkire J. (2020). COVID-19 pandemic, psychological response to quarantine, and knowledge of the disease among inmates in a Nigerian custodial center. Emerald Open Res..

[46] Montoya-Barthelemy A.G., Lee C.D., Cundiff D.R., Smith E.B. (2020). COVID-19 and the Correctional Environment: The American Prison as a Focal Point for Public Health. Am. J. Prev. Med..

[47] Brelje A.B., Pinals D.A. (2020). Provision of health care for prisoners during the COVID-19 pandemic: An ethical analysis of challenges and summary of select best practices. Int. J. Prison. Health.

[48] Winkelman T.N.A., Dasrath K.C., Young J.T., Kinner S.A. (2022). Universal health coverage and incarceration. Lancet Public Health.

[49] Tadros E., Aguirre N., Jensen S., Poehlmann-Tynan J. (2021). COVID-19 Inspired Relational Telemental Health Services for Incarcerated Individuals and Their Families. Contemp. Fam. Ther..

[50] Collica-Cox K., Molina L. (2020). A Case Study of the Westchester County New York’s Jail Response to COVID-19: Controlling COVID while Balancing Service Needs for the Incarcerated-A National Model for Jails. Vict. Offenders.

[51] Garau M.G.R., Martins I.M. (2020). Visitation in prison units in Rio de Janeiro: An analysis of the role of the family in the prison system from the pandemic. Captura Criptíca.

[52] Testa A., Fahmy C. (2021). Family member incarceration and coping strategies during the COVID-19 pandemic. Health Justice.

[53] Akiyama M.J., Spaulding A.C., Rich J.D. (2020). Flattening the Curve for Incarcerated Populations—COVID-19 in Jails and Prisons. N. Engl. J. Med..

[54] Tannuss R.W., Junior N.G.D.S.S., De Oliveira I.M.F.F. (2018). Pena compartilhada: Das relações entre cárcere, família e direitos humanos. Rev. Eletrônica Direito E Soc. REDES.

[55] Mezzina R., Gopikumar V., Jenkins J., Saraceno B., Sashidharan S.P. (2022). Social Vulnerability and Mental Health Inequalities in the “Syndemic”: Call for Action. Front. Psychiatry.

[56] LeMasters K., Ranapurwala S., Maner M., Nowotny K.M., Peterson M., Brinkley-Rubinstein L. (2022). COVID-19 community spread and consequences for prison case rates. PLoS ONE.

[57] Henry B.F. (2021). Reducing COVID-19 outbreaks in prisons through public health-centred policies. Lancet Public Health.

[58] Cloud D.H., Ahalt C., Augustine D., Sears D., Williams B. (2020). Medical Isolation and Solitary Confinement: Balancing Health and Humanity in US Jails and Prisons During COVID-19. J. Gen. Intern. Med..

[59] Lobel J., Smith P.S. (2019). Solitary Confinement: Effects, Practices, and Pathways toward Reform.

[60] Dalen K., Jones L. (2010). Ethical Monitoring: Conducting Research in a Prison Setting. Res. Ethic.

[61] Bosworth M., Campbell D., Demby B., Ferranti S.M., Santos M. (2005). Doing Prison Research: Views From Inside. Qual. Inq..

[62] Tsabedze W.F., Fourie E., Mhlanga S. (2023). Coping strategies of the incarcerated during the COVID-19 pandemic: A scoping review protocol of quantitative and qualitative evidence. BMJ Open.

[63] Chimicz D., Lewicka-Zelent A., Lisiecka A. (2023). Mood and Emotions among Inmates after COVID-19 Pandemic. Int. J. Environ. Res. Public Health.

